# Making Prodrugs Stick Around: Engineering Protease-Activated Tumor Retention

**DOI:** 10.1021/acscentsci.6c01098

**Published:** 2026-07-09

**Authors:** Robin Kryštůfek, Jan Konvalinka

**Affiliations:** Institute of Organic Chemistry and Biochemistry, Academy of Science of the Czech Republic, Flemingovo n. 2, 166 10 Praha 6, Czech Republic

## Abstract

Kang et al. combine protease-triggered activation with membrane trapping, keeping drugs in tumors longer and boosting delivery, imaging, and therapy.

## Graphical abstract

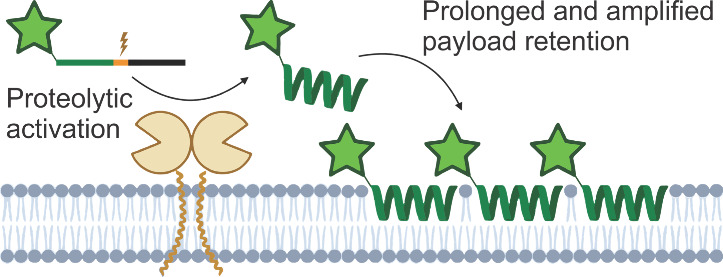

Protease-activated membrane tethering may help solve a major problem of targeted anticancer drug delivery: once a drug is released in the tumor, how do you keep it there? In a recent issue of *ACS Central Science*, the teams of Charly Craik and Michael Evans at UCSF introduce an interesting approach to tackle this challenge.^1^

For decades, anticancer drug delivery has been guided by a deceptively simple idea: make a drug inactive while it circulates, then activate it only where and when needed.^2^ This prodrug logic has inspired many elegant chemical designs that respond to low pH,^3^ redox gradients,^4^ hypoxia,^5^ or tumor-associated enzymes.^6,7^ Yet activation is only the first half of the delivery problem. Once a payload is released, it can diffuse away, be exported, or fail to accumulate long enough to produce a durable therapeutic effect. In solid tumors, where drug penetration, washout, stromal barriers, and heterogeneous biology all conspire against efficient delivery, “release” does not automatically mean “retention.”

In solid tumors, where drug penetration, washout, stromal barriers, and heterogeneous biology all conspire against efficient delivery, “release” does not automatically mean “retention.”

Kang et al. introduce an attractive answer to this problem: to couple enzymatic activation to local membrane trapping. Their platform, termed restricted interaction peptides, or RIPs, uses protease cleavage not merely as a switch to unmask a cargo, but as a trigger that exposes a membrane-binding domain (Figure 1). The result is a two-step mechanism: a disease-associated protease activates the construct, and the activated peptide then associates with nearby membranes, increasing the local payload residence at the site of proteolysis.

**Figure 1:**
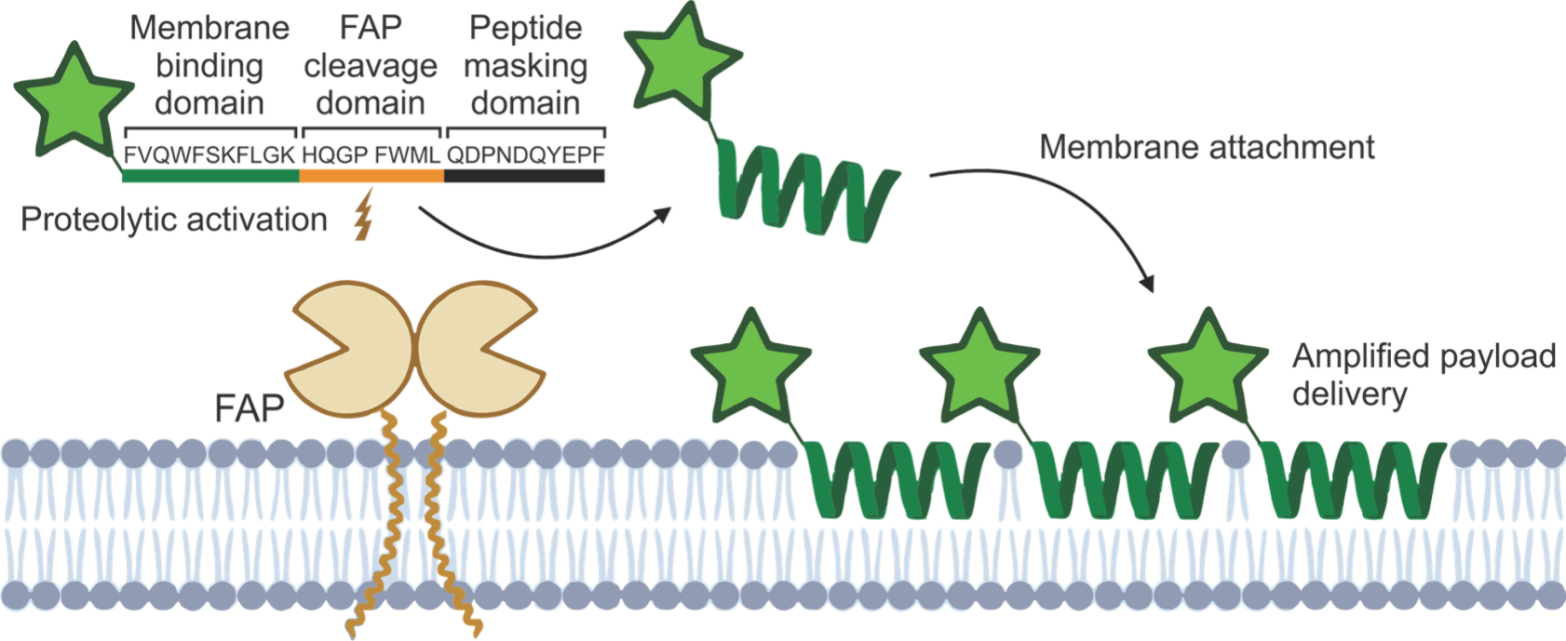
Schematic representation of the restricted-interaction peptide strategy, showing the sequence of the lead construct FRIP2. FAP-mediated cleavage at the cell membrane unmasks the amphipathic membrane-binding domain, enabling membrane engagement and amplifying payload delivery.

The authors focus on fibroblast activation protein, FAP, a cell-surface serine protease enriched in cancer-associated fibroblasts and fibrotic tissue, a target for active development of diagnostic and theranostic tools.^8^ FAP signals an activated stromal state associated with matrix remodeling, invasion, and immunosuppressive tumor microenvironments, making it an attractive target across many solid tumors while its expression in normal tissues is typically very low. However, the very success of FAP imaging has highlighted a therapeutic challenge: many active-site-directed FAP radioligands show impressive tumor uptake but insufficient retention for optimal radiotherapy. A catalytic activation-and-trapping approach offers a major advantage over stoichiometric receptor or enzyme binding. One FAP molecule can activate multiple RIP molecules, and each activated product can then be retained locally through membrane association.

To build FAP-activated RIPs, or FRIPs, the authors first used multiplex substrate profiling by mass spectrometry to identify efficient FAP-cleavable sequences. The best substrate sequence, HIGP-TAAY, was incorporated between a membrane-binding domain derived from Temporin L^9^ and a masking domain that suppresses nonspecific membrane interaction before the protease cleavage occurs. In cell experiments, fluorescent FRIP rapidly associated with and entered FAP-expressing U-251 cells, whereas FAP inhibition reduced membrane interaction. This is an important mechanistic demonstration because it shows that the peptide is cleaved and its hydrolysis is functionally connected to the membrane binding.

The therapeutic experiments then test whether this mechanism can improve targeting of real payloads. Conjugation of FRIP1 to the cytotoxin MMAE yielded FAP-dependent cytotoxicity in vitro. Inhibition or knockdown of FAP reduced the toxicity, while increasing FAP expression enhanced it. A control construct lacking the Val-Cit cathepsin-cleavable linker was much less potent, supporting the proposed sequence of events: FAP-dependent membrane interaction and uptake, followed by intracellular payload release. In mouse xenograft models, FRIP1-MMAE inhibited tumor growth more effectively than free MMAE while avoiding the severe weight loss observed with the free drug.

The remarkable success of protease-targeted radiotherapy of prostate carcinoma^10^ inspired extensive exploration of other specific cancer antigens, including FAP, as delivery addresses for radiochemicals. By attaching a chelating agent to FRIPs, the authors produced Cu-64 and Cu-67 labeled constructs for imaging and therapy. The FAP-optimized FRIP2, with substrate sequence HQGP-FWML, showed selective uptake in FAP-expressing tumors compared with FAP-low models, and it outperformed a granzyme-B-targeted RIP control. In a head-to-head comparison with radioligand targeted simply by a specific FAP inhibitor (Cu-67-FAPI-46), Cu-64-FRIP2 achieved substantially higher and more persistent tumor uptake despite similar blood clearance.

The broader significance of this work is that it reframes prodrug design around residence time, not just activation. A therapeutic payload with a protease-cleavable linker can answer the question “where is the drug released?”; however, a membrane-tethering motif begins to answer “where does the drug go next?” That second question is especially important for payloads whose efficacy depends on local concentration, internalization, or radiation dose deposition.

There are still important questions ahead. Membrane-active peptides can be sensitive to sequence, cargo, charge, and formulation, and their behavior may differ across tumor types, stromal architectures, and species. FAP is also expressed in some remodeling or fibrotic tissues, so therapeutic index will need careful clinical evaluation.^11^ For radioligand therapy, dosimetry, isotope choice, residence in the kidney and liver as well as tumor heterogeneity will be central. For cytotoxic conjugates, the balance between membrane trapping, internalization, bystander effects, and off-tumor activation will define the most suitable payloads.

The work therefore points beyond FAP alone toward a general design principle: use cancer microenvironment chemistry not only to switch drugs on, but to make them stay.

Still, the idea is powerful because it is modular. In principle, different protease-cleavable domains could be inserted to target distinct disease-associated enzymatic activities, while different payloads could be attached for imaging, chemotherapy, or radiotherapy. The work therefore points beyond FAP alone toward a general design principle: use cancer microenvironment chemistry not only to switch drugs on, but to make them stay.

## †

Department of Early Discovery Biochemistry, Genentech Inc., 1 DNA Way, South San Francisco, CA 94080, USA
